# Author Correction: In your phase: neural phase synchronisation underlies visual imagery of faces

**DOI:** 10.1038/s41598-021-94756-7

**Published:** 2021-08-02

**Authors:** Andrés Canales‑Johnson, Renzo C. Lanfranco, Juan Pablo Morales, David Martínez‑Pernía, Joaquín Valdés, Alejandro Ezquerro‑Nassar, Álvaro Rivera‑Rei, Agustín Ibanez, Srivas Chennu, Tristan A. Bekinschtein, David Huepe, Valdas Noreika

**Affiliations:** 1 Department of Psychology, University of Cambridge, Downing Site, Cambridge, CB2 3EB UK; 2 Vicerrectoría de Investigación y Posgrado, Universidad Católica del Maule, Talca, Chile; 3 Department of Psychology, University of Edinburgh, Edinburgh, UK; 4 Department of Neuroscience, Karolinska Institute, Stockholm, Sweden; 5 Facultad de Psicología, Pontificia Universidad Católica de Chile, Santiago, Chile; 6 Escuela de Psicología, Universidad Adolfo Ibáñez, Santiago, Chile; 7 School of Computing, University of Kent, Chatham Maritime, UK; 8 Department of Clinical Neurosciences, University of Cambridge, Cambridge, UK; 9 Department of Biological and Experimental Psychology, School of Biological and Chemical Sciences, Queen Mary University of London, London, UK; 10 Center for Social and Cognitive Neuroscience (CSCN), Latin American Institute of Brain Health (BrainLat), Universidad Adolfo Ibanez, Santiago, Chile; 11 National Scientific and Technical Research Council (CONICET), Buenos Aires, Argentina; 12 Universidad Autónoma del Caribe, Barranquilla, Colombia; 13 Cognitive Neurosience Center (CNC), Universidad de San Andrés, Buenos Aires, Argentina; 14 Global Brain Health Institute (GBHI), University of California San Francisco (UCSF), San Francisco, USA

Correction to: *Scientific Reports* 10.1038/s41598-021-81336-y, published online 27 January 2021

This Article contained an error in the Figure legends of Figure 3 and Figure 4. The legends of these Figures were inadvertently switched.

The legend of Figure 3:

“Gamma phase-synchrony between occipital and parietal electrodes. wPLI (30–60 Hz) for visual imagery (**A**) and control (**B**) conditions. (**C**) Cluster-based permutation test comparing visual imagery and control conditions. The area highlighted in the time–frequency chart depicts a significant wPLI cluster between conditions (visual imagery minus control) in the gamma band (30–60 Hz). (**D**) Region of interest (ROI) for phase-synchrony analysis. wPLI values are expressed in standard deviations (z-scores) in reference to the baseline (− 1500 to − 1250 ms). Trial length (− 1500 ms) is relative to response time (0 ms). (**D**) Topographical representation of occipitoparietal wPLI channel pairs and single-participant wPLI values for the imagery (left) and control (right) conditions. Each arc represents a pair of channels, and the height of the arc is its normalised value. Grey circles represent single-participant wPLI values for the cluster depicted in (**C**) for the imagery (left panel) and control (right panel) conditions. The red horizontal line represents the group mean, the rectangle represents SEM, and the red-dashed horizontal line represents the group median.”

now reads:

“Theta phase-synchrony between inter-hemispheric frontal electrode pairs. wPLI (1–10 Hz) for visual imagery (**A**) and control (**B**) conditions. (**C**) Cluster-based permutation test comparing visual imagery and control conditions. The area highlighted in the time–frequency chart depicts a significant wPLI cluster between conditions (visual imagery minus control) in the theta band (5–7 Hz). (**D**) Region of interest (ROI) for phase-synchrony analysis. Only interhemispheric frontofrontal pairs of channels were considered for the analysis (see Sect. 2.8 for details). wPLI values are expressed in standard deviations (z-scores) in reference to the baseline (− 1500 to − 1250 ms). Trial length (− 1500 ms) is relative to response time (0 ms). (**D**) Topographical representation of frontofrontal wPLI channel pairs and single-participant wPLI values for the imagery (left) and control (right) conditions. Each arc represents a pair of channels, and the height of the arc is its normalised value. Grey circles represent single-participant wPLI values for the cluster depicted in (**C**) for the imagery (left panel) and control (right panel) conditions. The red horizontal line represents the group mean, the rectangle represents SEM, and the red-dashed horizontal line represents the group median.”

The legend of Figure 4:

“Theta phase-synchrony between inter-hemispheric frontal electrode pairs. wPLI (1–10 Hz) for visual imagery (**A**) and control (**B**) conditions. (**C**) Cluster-based permutation test comparing visual imagery and control conditions. The area highlighted in the time–frequency chart depicts a significant wPLI cluster between conditions (visual imagery minus control) in the theta band (5–7 Hz). (**D**) Region of interest (ROI) for phase-synchrony analysis. Only interhemispheric frontofrontal pairs of channels were considered for the analysis (see Sect. 2.8 for details). wPLI values are expressed in standard deviations (z-scores) in reference to the baseline (− 1500 to − 1250 ms). Trial length (− 1500 ms) is relative to response time (0 ms). (**D**) Topographical representation of frontofrontal wPLI channel pairs and single-participant wPLI values for the imagery (left) and control (right) conditions. Each arc represents a pair of channels, and the height of the arc is its normalised value. Grey circles represent single-participant wPLI values for the cluster depicted in (**C**) for the imagery (left panel) and control (right panel) conditions. The red horizontal line represents the group mean, the rectangle represents SEM, and the red-dashed horizontal line represents the group median.”

now reads:

“Gamma phase-synchrony between occipital and parietal electrodes. wPLI (30–60 Hz) for visual imagery (**A**) and control (**B**) conditions. (**C**) Cluster-based permutation test comparing visual imagery and control conditions. The area highlighted in the time–frequency chart depicts a significant wPLI cluster between conditions (visual imagery minus control) in the gamma band (30–60 Hz). (**D**) Region of interest (ROI) for phase-synchrony analysis. wPLI values are expressed in standard deviations (z-scores) in reference to the baseline (− 1500 to − 1250 ms). Trial length (− 1500 ms) is relative to response time (0 ms). (**D**) Topographical representation of occipitoparietal wPLI channel pairs and single-participant wPLI values for the imagery (left) and control (right) conditions. Each arc represents a pair of channels, and the height of the arc is its normalised value. Grey circles represent single-participant wPLI values for the cluster depicted in (**C**) for the imagery (left panel) and control (right panel) conditions. The red horizontal line represents the group mean, the rectangle represents SEM, and the red-dashed horizontal line represents the group median.”

This error has now been corrected in the PDF and HTML versions of the Article.

